# Comparison of methods for the analysis of therapeutic immunoglobulin G Fc-glycosylation profiles—Part 1: Separation-based methods

**DOI:** 10.4161/19420862.2014.986000

**Published:** 2014-12-18

**Authors:** Dietmar Reusch, Markus Haberger, Bernd Maier, Maria Maier, Ronny Kloseck, Boris Zimmermann, Michaela Hook, Zoltan Szabo, Samnang Tep, Jo Wegstein, Nadja Alt, Patrick Bulau, Manfred Wuhrer

**Affiliations:** 1 Pharma Biotech Development Penzberg; Roche Diagnostics GmbH; Penzberg, Germany; 2 ProZyme, Inc.; Hayward, CA USA; 3 Center for Proteomics and Metabolomics; Leiden University Medical Center; Leiden, The Netherlands; 4 Division of BioAnalytical Chemistry; Department of Chemistry and Pharmaceutical Sciences; VU University Amsterdam; Amsterdam, The Netherlands

**Keywords:** IgG glycosylation, monoclonal antibody (mAb), HILIC-UPLC, 2-AB labeling, APTS labeling, method comparison, DNA analyzer, HPAEC, glycan analysis, CE-LIF, high-throughput

## Abstract

Immunoglobulin G (IgG) crystallizable fragment (Fc) glycosylation is crucial for antibody effector functions, such as antibody-dependent cell-mediated cytotoxicity, and for their pharmacokinetic and pharmacodynamics behavior. To monitor the Fc-glycosylation in bioprocess development, as well as product characterization and release analytics, reliable techniques for glycosylation analysis are needed. A wide range of analytical methods has found its way into these applications. In this study, a comprehensive comparison was performed of separation-based methods for Fc-glycosylation profiling of an IgG biopharmaceutical. A therapeutic antibody reference material was analyzed 6-fold on 2 different days, and the methods were compared for precision, accuracy, throughput and other features; special emphasis was placed on the detection of sialic acid-containing glycans. Seven, non-mass spectrometric methods were compared; the methods utilized liquid chromatography-based separation of fluorescent-labeled glycans, capillary electrophoresis-based separation of fluorescent-labeled glycans, or high-performance anion exchange chromatography with pulsed amperometric detection. Hydrophilic interaction liquid chromatography-ultra high performance liquid chromatography of 2-aminobenzamide (2-AB)-labeled glycans was used as a reference method. All of the methods showed excellent precision and accuracy; some differences were observed, particularly with regard to the detection and quantitation of minor glycan species, such as sialylated glycans.

## Abbreviations

mAb: monoclonal antibody
Fc: fragment crystallizable
IgG: immunoglobulin G
HILIC-UPLC: hydrophilic interaction liquid chromatography-ultra high performance liquid chromatography
2-AB: 2-aminobenzamide
Fab: fragment, antigen-binding
CE-LIF: capillary electrophoresis-laser induced fluorescence
HPLC: high performance liquid chromatography
MALDI-MS: matrix-assisted laser desorption/ionization-mass spectrometry
ESI-MS: electrospray ionization-mass spectrometry
HPAEC-PAD: high-performance anion exchange chromatography with pulsed amperometric detection
APTS: 8-aminopyrene-1, 3, 6-trisulfonic acid
DSA-FACE: DNA-sequencer-aided fluorophore-assisted carbohydrate electrophoresis
ANTS: 8-aminonaphthalene-1, 3, 6-trisulfonate
CCGE: cartridge-based capillary gel electrophoresis
HR: high resolution
IAB: InstantAB labeling
CHO: Chinese hamster ovary

## Introduction

Recombinant monoclonal antibodies (mAbs) are efficacious therapeutic agents for various disease areas, including inflammatory and autoimmune diseases as well as cancer.^1,2^ The use of mammalian expression systems results in a remarkable heterogeneity of mAb products, generally due to post-translational modifications, such as N- and C-terminal modifications, deamidation, isomerization and glycosylation. Glycosylation is a critical post-translational modification because it may affect mAbs characteristics such as solubility, stability, pharmacokinetic and pharmacodynamics properties, as well as in vivo efficacy.^3,4^

Currently, therapeutic mAbs are almost exclusively the IgG isotype, and bear in their Fc domain an N-glycan chain linked to asparagine 297 (numbering according to Kabat).^5^ Depending on the cellular expression system, these N-glycans are a mixture of complex-, hybrid- or high mannose-type glycans. The main part is normally composed of fucosylated complex-type biantennary oligosaccharides that may lack a core fucose and may include a bisecting N-acetylglucosamine. Additionally, they vary in galactose and sialic acid content.^4,6-8^

Some therapeutic mAbs may carry additional N-glycans in the variable regions of the antigen-binding (Fab) domain and these glycans may affect antigen binding.^9,10^

The glycosylation pattern has a great impact on the antibody effector functions. A relatively high amount of galactose may result in activation of the complement system and, by IgG binding to C1q, results in complement-dependent cellular cytotoxicity.^11^ Afucosylation (lack of core fucose) promotes antibody-dependent cellular cytotoxicity (ADCC) by increased binding of the IgG Fc portion to FcγRIIIa on natural killer cells.^12,13^

Glycoforms can also affect pharmacodynamics and pharmacokinetics. Recent studies described clear evidence for selective clearance of oligomannose species (high mannose-type) of Fc glycans.^14,15^ Sialylation may induce anti-inflammatory effects via Th2 signaling and decrease ADCC via reduced interaction with Fcγ receptors.^16,17^ Additionally, some IgG glycan structures such as α1,3-bound galactose and N-glycolylneuraminic acid may be involved in adverse immune reactions.^18,19^ Due to its various functional implications, the glycosylation pattern of a therapeutic antibody may represent a critical quality attribute, and therefore may require close monitoring during bioprocess development and routine manufacturing.^20^ A wide range of state-of-the-art analytical methods to monitor Fc-glycosylation is available. In principle the methods can be sub-divided into 3 categories:^3,21–23^ (1) Analysis of the IgG molecule with electrospray ionization mass spectrometry (ESI-MS)—either on the intact molecule after reduction of disulphide bonds, or after a limited digestion with a proteolytic enzyme and deduction of the overall glycan composition;^24–26^ (2) Enzymatic release of the Fc glycans and measurement with mass spectrometric methods, by HPLC with pulsed amperometric detection or by capillary electrophoresis (CE)/HPLC-based methods after fluorescent labeling;^27,28^ and (3) Proteolytic cleavage of the IgG molecule and analysis of the glycopeptides with matrix-assisted laser desorption/ionization-mass spectrometry (MALDI-MS) or electrospray ionization-mass spectrometry ESI-MS.^29–32^

Comparisons of different methods for analysis of IgG Fc-glycosylation have been reported, but these studies included a limited number of methods or compared mainly mass spectrometry-based methods.^33–38^ Thus, a thorough comparison of different methods for glycoanalysis is still lacking.

As a consequence, we performed an extensive study on both non-mass spectrometric and mass spectrometric methods for IgG Fc-glycosylation analysis. The study involved 3 laboratories: a biopharmaceutical company (Roche Diagnostics), an academic research laboratory (Leiden University Medical Center) and a vendor of tools for glycan analysis (ProZyme, Inc.). The same mAb sample was analyzed 6-fold on 2 different days. Special attention was paid to the measurement of low levels of sialylation. Methods were compared with regard to separation power, precision, accuracy, required resources and throughput. Due to its wide acceptance, hydrophilic interaction liquid chromatography-ultra high performance liquid chromatography (HILIC-UHPLC) of N-glycans after labeling with 2-AB was used as the reference method against which the other methods were compared [HILIC(2-AB), the Reference Method].^22^ The entire study has been divided into 2 parts; the first part, comparing the non-mass spectrometric methods, is presented here.

## Results

In this study, 7 non-mass spectrometric, separation-based methods (summarized in **Table 1**) were evaluated for the analysis of the Fc-glycosylation of an IgG1 monoclonal antibody (mAb1). One method relied on electrochemical detection of native, released glycans after separation at high pH: high-performance anion exchange chromatography with pulsed amperometric detection (HPAEC-PAD).^28,39–42^ All other methods applied fluorescent labeling. Four methods used electrophoretic separation, which included conventional high-resolution capillary gel electrophoresis with laser-induced fluorescence [CE-LIF(APTS-HR1)],^43–48^ DNA-sequencer-aided fluorophore-assisted carbohydrate electrophoresis after 8-aminopyrene-1,3,6-trisulfonic acid (APTS) labeling for high-throughput screening [DSA-FACE(APTS)],^49–54^ high-resolution capillary gel electrophoresis with rapid labeling with APTS via reductive amination [CE-LIF(APTS-HR2)], and cartridge-based capillary gel electrophoresis with rapid 8-aminonaphthalene-1,3,6-trisulfonate (ANTS) labeling, in development specifically for screening [CCGE(ANTS)]. The CE methods differ with regard to the labeling method: APTS labeling for the “normal” method requires 4 to 24 h for labeling, whereas rapid reductive amination has been optimized to yield unbiased labeling in 1 h. Another difference lies in the type of label used: 3 methods used APTS, and one ANTS. Different CE hardware systems were also used: CE-LIF(APTS-HR1) and CE-LIF(APTS-HR2) were analyzed on a Beckman Coulter 800 *plus* Pharmaceutical Analysis System; DSA-FACE(APTS) was analyzed on an Applied Biosystems ABI 3730xl DNA Analyzer; and CCGE(ANTS) was analyzed on ProZyme's Merlin Cartridge-based Capillary Gel Electrophoresis System.

**Table 1.** Overview of used methods

Method	Description
HILIC(2-Ab)/The Reference Method	2-AB labeling of released glycans; separation with HILIC-UPLC
HILIC(IAB)	Labeling of released glycans with InstantAB; separation with HILIC-HPLC
CE-LIF(APTS-HR1)	APTS-labeling of released glycans and separation with CE
DSA-FACE(APTS)	DSA-FACE employing APTS-labeling of released glycans, separation with multiplexing CGE-LIF
CE-LIF(APTS-HR2)	Labeling of released glycans with Rapid- Reductive-Amination APTS; separation with capillary electrophoresis
CCGE(ANTS)	ANTS-labeling of released glycans with Rapid-Reductive-Amination ANTS; separation with cartridge-based capillary gel electrophoresis
HPAEC-PAD	Separation with high pH anion exchange HPLC; detection with pulsed amperometric detection

The 2 remaining chromatographic methods used HILIC separation of labeled glycans, with HILIC profiling of 2-AB-labeled glycans, one serving as the Reference Method and the other using InstantAB labeling [HILIC(IAB)].^55–59^ Both were analyzed on a Waters ACQUITY UPLC® System (or a Dionex RSLC Ultimate 3000RS).

Three laboratories were involved in performing the experiments, an analytical laboratory in a development department, a quality control laboratory and a laboratory of a vendor of glycoanalytical tools.

### Peak assignment

Peak assignment of the 2 HILIC-based methods for analyzing glycans, [HILIC(2-AB) and HILIC(IAB)], was accomplished by online coupling of HILIC-UHPLC with ESI-MS. For isomeric glycan structures, as well as for confirmation of the mass spectrometric results, the elution position relative to a hexose homopolymer standard (glucose units) was taken into account for structural assignment.^33^ For HPAEC-PAD, peaks were identified by spiking commercially available glycan standards and by employing exoglycosidase digests. Additionally the peaks were confirmed by online desalting and coupling to ESI-MS.^60^ For CE-LIF(APTS-HR1), peaks were assigned by online coupling to ESI-MS as described by Gennaro et al.^43^

For DSA-FACE(APTS), assignment of peaks is described in Reusch et al.^53^ Briefly, glycan identification relied on the use of commercially available glycan standards (after APTS labeling) used to spike the DSA-FACE(APTS) analysis of APTS-labeled mAb1 glycans after HILIC-UHPLC fractionation. In addition, online ESI-MS(/MS) coupling of the HILIC-UHPLC separation of the APTS-labeled mAb1 N-glycans was employed for further structural elucidation.

The peak assignment for CE-LIF(APTS-HR2) and CCGE(ANTS) was accomplished by spiking commercially available glycan standards and by relying on the well-known order of elution of the different glycans.^33^

### Detected glycosylation features

**Figure 1.**
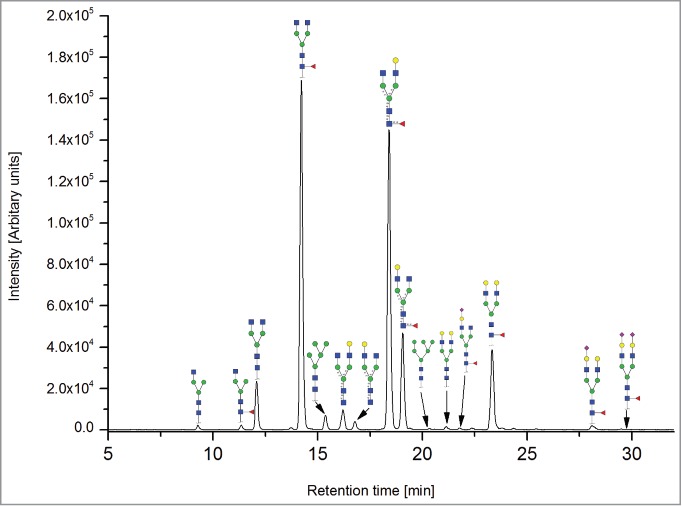
HILIC-UPLC of 2-AB-labeled N-glycans [HILIC(2-AB), the Reference Method]. Key: blue square, N-acetylglucosamine; green circle, mannose; yellow circle, galactose; red triangle, fucose; purple diamond, N-acetylneuraminic acid.

**Figure 2.**
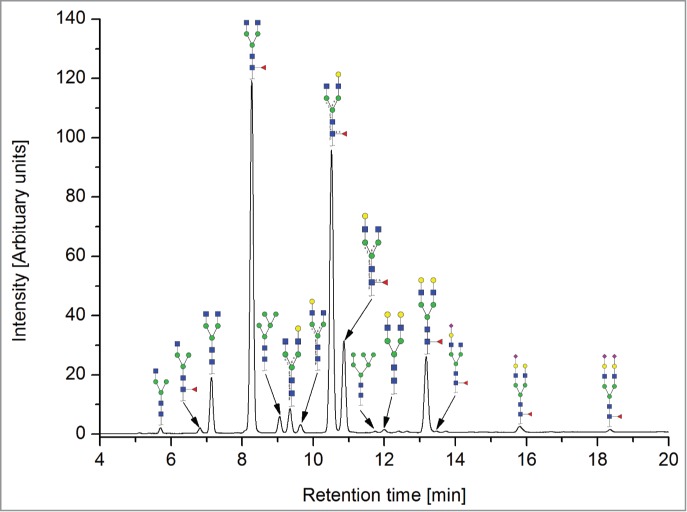
HILIC-UPLC of N-glycans labeled with InstantAB [HILIC)IAB)]. For key, see **Figure 1**.

All the methods facilitated separation of the main Fc N-glycan species that are typically found on therapeutic IgG mAbs produced in Chinese hamster ovary (CHO) cells (G0F, G1F, G2F, G0, G1 and M5; see **Table 2** for key). The Reference Method allowed the resolution and quantitation of 15 glycan species (**Fig. 1**). The related HILIC(IAB) method showed a similar resolution and likewise allowed the detection of 15 glycan structures (**Fig. 2**).

**Table 2.** Quantitative evaluation of method performance. Each glycoanalytical method was applied in 2 series (batches), 6 replicates per batch. Relative abundance of the various glycan species are given in percent, with standard deviations in parentheses. For G1F(1,6), fucosylated, monogalactosylated biantennary glycan with galactosylation of the 1,6-arm, the percentage within the overall G1F species is given in brackets. Key: H, hexose; N, N-acetylhexosamine; F, deoxyhexose; S, N-acetylneuraminic acid (sialic acid); G0F-N, agalacosylated, core-fucosylated, monoantennary species, *etc*; n.d.: not detected; n.a.: not applicable.

			HILIC(2-AB) Reference Method		HILIC(IAB)		CE-LIF(APTS-HR1)		DSA-FACE(APTS)		CE-LIF(APTS-HR2)		CCGE(ANTS)		HPAEC-PAD	
Glycan species No.	Short name [composition]	Structurascheme	1. series	2. series	1. series	2. series	1. series	2. series	1. series	2. series	1. series	2. series	1. series	2. series	1. series	2. series
1	G0F [H3N4F1]	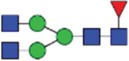	35.5 (0.1)	35.3 (0.1)	33.5 (0.4)	34.1 (0.7)	36.1 (0.1)	36.0 (0.2)	36.7 (0.4)	35.7 (0.6)	34.6 (0.1)	34.6 (0.2)	36.0 (0.4)	36.2 (0.2)	37.7 (0.3)	37.0 (0.8)
2+3	G1F [H4N4F1]	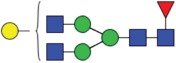	43.4 (n.a.)	43.3 (n.a.)	42.8 (n.a.)	42.9 (n.a.)	45.3 (n.a.)	45.2 (n.a.)	44.3 (n.a.)	44.6 (n.a.)	43.6 (n.a.)	43.5 (n.a.)	44.4 (n.a.)	44.9 (n.a.)	43.6 (n.a.)	43.6 (n.a.)
2	G1F(1,6) [H4N4F1]	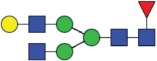	32.7 (0.1) [75.3]	32.6 (0.1) [75.3]	32.3 (0.1) [75.5]	32.4 (0.2) [75.6]	34.4 (<0 .1) [75.9]	34.4 (0.1) [76.1]	33.6 (0.2) [75.8]	33.5 (0.7) [75.1]	33.6 (0.1) [77.0]	33.4 (0.1) [76.7]	33.2 (0.3) [74.8]	33.4 (0.2) [74.4]	33.3 (0.4) [76.4]	33.9 (0.5) [77.7]
3	G1F(1,3) [H4N4F1]	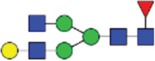	10.8 (<0 .1)	10.7 (<0 .1)	10.6 (0.3)	10.5 (0.1)	10.9 (0.1)	10.7 (0.1)	10.7 (0.2)	11.1 (1.0)	10.1 (0.1)	10.2 (0.1)	11.3 (0.2)	11.4 (0.1)	10.3 (0.1)	9.7 (0.3)
4	G2F [H5N4F1]	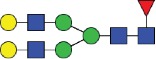	9.5 (<0 .1)	9.6 (0.1)	10.6 (0.3)	10.2 (0.4)	9.4 (0.1)	9.4 (<0 .1)	9.0 (0.2)	9.5 (0.1)	10.1 (0.1)	10.2 (0.1)	9.1 (0.1)	9.2 (0.1)	8.3 (0.2)	8.2 (0.3)
5	G0 [H3N4]	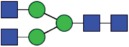	4.6 (0.1)	4.7 (0.1)	4.5 (0.1)	4.6 (0.1)	3.7 (0.1)	3.8 (<0 .1)	4.1 (0.3)	4.2 (0.2)	4.7 (<0 .1)	4.7 (<0 .1)	3.9 (0.3)	3.6 (<0 .1)	4.1 (0.1)	4.8 (0.2)
6+7	G1 [H4N4]	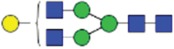	3.3 (n.a.)	3.4 (n.a.)	3.3 (n.a.)	3.3 (n.a.)	2.4 (n.a.)	2.4 (n.a.)	2.3 (n.a.)	2.8 (n.a.)	2.9 (n.a.)	2.9 (n.a.)	2.5 (0.1)	2.4 (0.1)	2.2 (n.a.)	2.3 (n.a.)
6	G1 1,6 [H4N4]	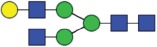	2.4 (<0 .1)	2.4 (<0 .1)	2.4 (<0 .1)	2.4 (<0 .1)	1.6 (0.1)	1.4 (<0 .1)	1.6 (0.2)	1.6 (0.1)	2.0 (<0 .1)	2.0 (<0 .1)	1.8 (0.1)	1.8 (0.1)	1.6 0.2	1.7 (0.1)
7	G1 1,3 [H4N4]	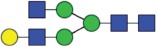	0.9 (<0 .1)	0.9 (<0 .1)	0.9 (<0 .1)	0.9 (<0 .1)	0.9 (0.1)	0,9 (<0 .0)	0.8 (0.1)	1.3 (0.1)	0.9 (<0 .1)	0.8 (<0 .1)	0.7 (0.1)	0.8 (<0 .1)	0.6 (0.1)	0.7 (0.1)
8	G2 [H5N4]	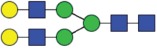	0.3 (<0 .1)	0.4 (<0 .1)	0.7 (<0 .1)	0.6 (<0 .1)	n.d.	n.d.	0.4 (0.1)	0.3 (<0 .1)	0.3 (<0 .1)	0.3 (<0 .1)	0.3 (<0 .1)	0.3 (<0 .1)	0.4 (0.1)	0.4 (0.1)
9	G0F-N [H3N3F1]	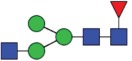	0.5 (<0 .1)	0.5 (<0 .1)	0.5 (<0 .1)	0.5 (<0 .1)	n.d.	n.d.	n.d.	n.d.	0.4 (<0 .1)	0.4 (<0 .1)	n.d.	n.d.	0.3 (0.1)	0.3 (<0 .1)
10	G1F-N [H4N3F1]	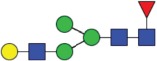	n.d.	n.d.	n.d.	n.d.	n.d.	n.d.	n.d.	n.d.	n.d.	n.d.	n.d.	n.d.	0.5 (0.1)	0.5 (0.1)
11	G0-N [H3N3]	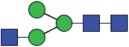	0.4 (<0 .1)	0.4 (<0 .1)	0.4 (<0 .1)	0.4 (<0 .1)	0.2 (<0 .1)	0.3 (<0 .1)	0.4 (0.1)	0.4 (<0 .1)	0.4 (<0 .1)	0.4 (<0 .1)	n.d.	n.d.	0.5 (0.1)	0.5 (0.1)
12	M5 [H5N2]	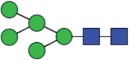	1.5 (<0 .1)	1.6 (<0 .1)	1.5 (<0 .1)	1.5 (<0 .1)	2.1 (0.1)	2.2 (<0 .1)	1.7 (0.2)	1.8 (0.1)	2.2 (0.1)	2.2 (<0 .1)	1.9 (0.1)	1.7 (0.1)	1.5 (0.1)	1.6 (0.2)
13	M6 [H6N2]	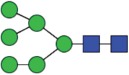	0.1 (<0 .1)	0.1 (<0 .1)	0.2 (<0 .1)	0.2 (0.2)	n.d.	n.d.	n.d.	n.d.	n.d.	n.d.	n.d.	n.d.	0.1 (0.1)	0.1 (<0 .1)
14	G1FS [H4N4F1S1]	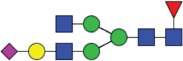	0.2 (<0 .1)	0.2 (<0 .1)	0.2 (<0 .1)	0.2 (<0 .1)	0.3 (<0 .1)	0.3 (<0 .1)	0.6 (0.1)	0.5 (0.1)	0.3 (<0 .1)	0.3 (<0 .1)	n.d.	n.d.	0.1 (0.1)	0.1 (<0 .1)
15	G2S1F [H5N4F1S1]	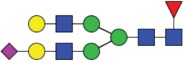	0.7 (<0 .1)	0.7 (0.1)	1.2 (<0 .1)	1.1 (0.1)	n.d.	n.d.	n.d.	n.d.	n.d.	n.d.	1.4 (0.1)	1.3 (0.1)	0.5 (<0 .1)	0.5 (0.1)
16	G2S2F [H5N4F1S2]	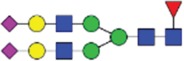	0.1 (<0 .1)	0.1 (<0 .1)	0.5 (<0 .1)	0.4 (<0 .1)	0.5 (<0 .1)	0.5 (<0 .1)	0.5 (0.2)	<0 .1 (<0 .1)	0.4 (<0 .1)	0.5 (<0 .1)	0.5 (0.1)	0.4 (<0 .1)	0.1 (0.1)	0.1 (<0 .1)

**Figure 3.**
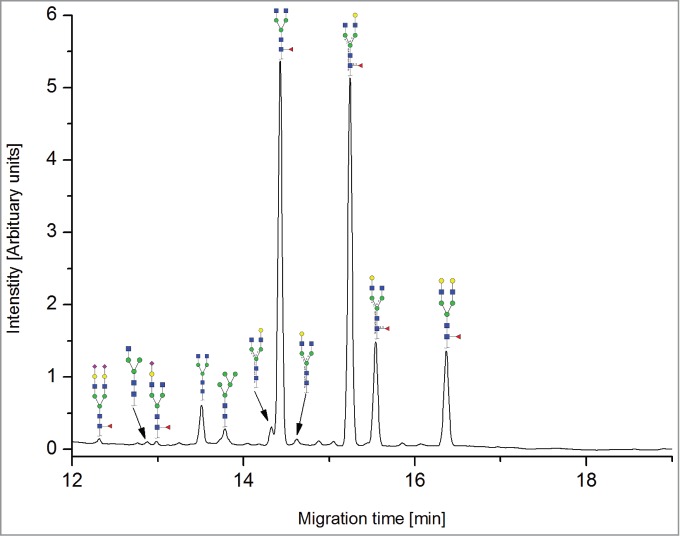
Capillary electrophoresis separation of APTS-labeled N-glycans with laser-induced fluorescence detection [CE-LIF(APTS-HR1)]. For key, see **Figure 1**.

**Figure 4.**
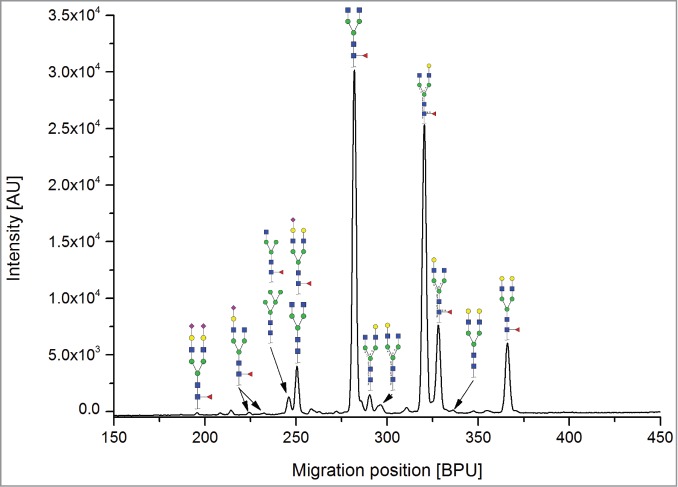
Multiplexing CGE-LIF analysis of APTS-labeled N-glycans on a DNA sequencer [DNA-sequencer-aided fluorophore-assisted carbohydrate electrophoresis; DSA-FACE(APTS)]. For key, see **Figure 1**.

**Figure 5.**
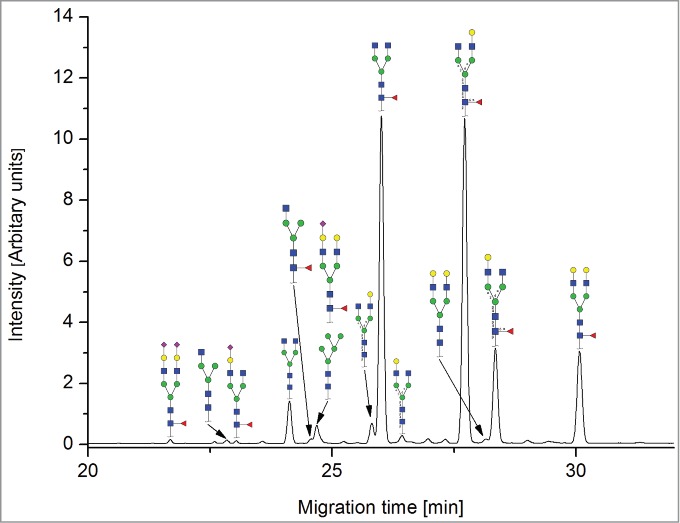
Capillary electrophoresis separation with laser-induced fluorescence detection of N-glycans labeled by rapid reductive amination [CE-LIF(APTS-HR2)]. For key, see **Figure 1**.

**Figure 6.**
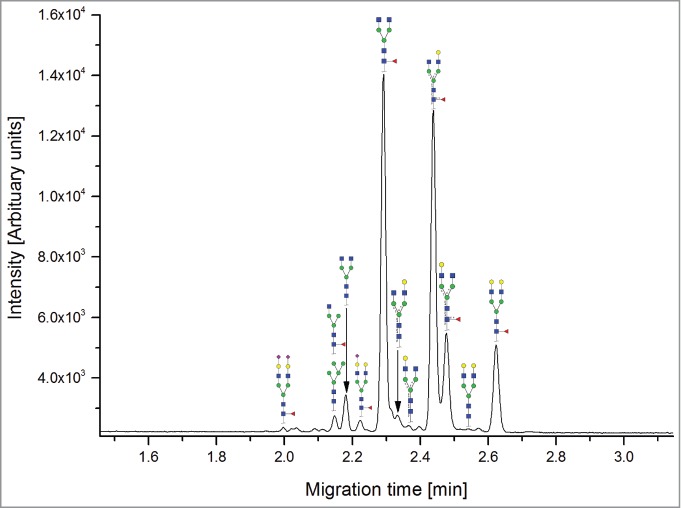
Capillary electrophoresis separation with laser-induced fluorescence detection of N-glycans labeled by rapid reductive amination [CCGE(Rapid Reductive Amination ANTS)]. For key, see **Figure 1**.

The CE methods showed a total number of detected glycan species between 11 and 14 (**Figs. 3, 4, 5** and **6**). HPAEC-PAD showed an excellent coverage of the mAb1 N-glycan species with 16 glycan species resolved and assigned.

Remarkably, all 7 methods allowed differentiation of isomers arising from upper (α1,6)- *vs*. lower (α1,3)-arm galactosylation of biantennary glycans, both with and without core-fucosylation [G1F(1,6); G1F(1,3); G1(1,6) and G1(1,3)]. The peak assignment of the monogalactosylated species was deduced from literature data: the peaks with upper (α1,6)-arm galactosylation were found for all methods to elute prior to the peaks with lower (α1,3)-arm galactosylation.^61–66^

Both HILIC-based methods and HPAEC-PAD also separated 3 sialic acid-containing glycans, namely G1FS [H4N4F1S1], G2S1F [H5N4F1S1] and G2S2F [H5N4F1S2]. All 4 CE-based methods separated 2 out of 3 of the sialylated species: CE-LIF(APTS-HR1), CE-LIF(APTS-HR2) and DSA-FACE(APTS) showed 2 resolved sialic acid-containing peaks (G1S1F and G2S2F; **Figs. 3, 4** and **5**), while the G2S1F co-migrated with M5 and G0. CCGE(ANTS) detected G2S1F and G2S2F (**Fig. 6**).

The high-mannose structure M5 [H5N2] was separated and quantified with all methods, whereas M6 [H6N2] was only detected with the 2 HILIC-based methods and HPAEC-PAD.

**Figure 7.**
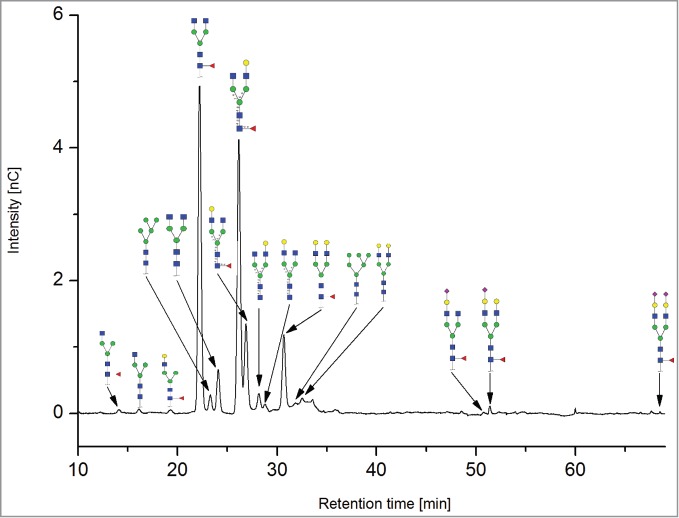
High-performance anion-exchange chromatography with pulsed amperometric detection (HPAEC-PAD) for the separation and detection of native N-glycans. For key, see **Figure 1**.

For mono-antennary structures (structures lacking an N-acetylglucosamine, such as G0F-N [H3N3F1], G1F-N [H4N3F1] and G0-N [H3N3] see **Table 2**), the separation capabilities were more diverse: G0F-N was separated with the 2 HILIC-based methods, with HPAEC-PAD and by CE-LIF(APTS-HR2), but not with CE-LIF(APTS-HR1) or CCGE(ANTS). Remarkably, G1F-N was only separated and identified by HPAEC-PAD (see **Fig. 7**).

The HILIC- based methods were able to separate nearly all species; only the minor species G1F-N [H4N3F1] co-eluted with G1(1,6) [H4N4]. Since G1F-N is generally found in CHO-derived mAbs at very low levels (<1 %), this co-migration is regarded as being non-critical. In general, the glycan peaks are very well separated, resulting in excellent quantitation performance (**Table 2**).

### The G0-N structure was able to be separated with all methods except CCGE(ANTS)

Other low-abundance glycan structures could also be separated. With the Reference Method, these minor structures were found, but for simplicity they were not included in **Table 2**: the mono-antennary structures G1-N [H4N3], M3 [H3N2] and M4 [H4N2]. HPAEC-PAD was the only method capable of separating all peaks included in the quantitation study. However, the baseline appears not to be as stable as for the HILIC- based method, impeding the peak integration for quantification.

Peak assignment for CE-based methods is more difficult because the instrument cannot easily be directly coupled to mass spectrometers without adapting the method. In addition, loading capacity of CE is relatively low, mainly due to low injection volumes, thereby complicating the coupling to mass spectrometric systems.

CE-LIF(APTS-HR1) and CE-LIF(APTS-HR2) were able to detect and quantify 11 peaks, but G2 and G0F-N could not be separated. DSA-FACE(APTS) separated 12 peaks that could be assigned, but some structures, G2S1F/G0 and M5/G0F-N, were found to be co-eluting. CCGE(ANTS) separated 11 peaks, missing G0F-N, G1F-N, G0-N, M6 and G1FS.

### Method performance with regard to accuracy and precision

A summary of the quantitative methods evaluation is shown in **Table 2**. HILIC(2-AB), being our reference method, showed excellent precision with low standard deviations between each series (consisting of 6 replicates), and only minute differences in average relative abundance were observed between the 2 series analyzed on different days. The other methods tested, with the exception of the CE-LIF(APTS-HR1), showed a slightly greater difference between the mean results on different days. Nevertheless, the results obtained on different days were comparable, with the inter-day differences in relative intensities of all glycan species below 1% for all 7 methods. For the major glycan species, relative abundances determined with the various methods were in good agreement with values obtained by the Reference Method. The G0F species average 35.4% relative abundance for HILIC(2-AB), while this value was found to be slightly higher for CE-LIF(APTS-HR1), DSA-FACE(APTS) and HPAEC-PAD, ranging from 36.0% to 37.7%, and slightly lower for HILIC(IAB), CCGE(ANTS) and CE-LIF(APTS-HR2), ranging from 33.5% to 34.6%. The 2 isomeric G1F species were found with a combined average relative abundance of 43.4% with HILIC(2-AB). This value was again found to be slightly higher for CE-LIF(APTS-HR1), DSA-FACE(APTS) and HPAEC-PAD, ranging from 43.6% to 45.3%, and slightly lower for HILIC(IAB), CCGE(ANTS) and CE-LIF(APTS-HR2), ranging from 42.4% to 43.0%. The relative amounts of the upper (α1,6)- *vs*. lower (α1,3)-arm galactosylation was similar for all methods employed, with the portion of G1F(1,6) of the total G1F ranging from 74.4% to 77.7% for all 7 methods and 2 time points (**Table 2**). For HILIC(2-AB), the relative abundance of fucosylated mono-galactosylated species was 32.7% (α1,6) and 10.8% (α1,3), respectively, which was in good agreement with all other methods, ranging from 31.71% to 34.4% for the α1,6 variant, and 9.7% to 11.4% for the α1,3 variant. The same holds true for non-fucosylated glycan species, where the relative amounts of the upper (α1,6)- *vs*. lower (α1,3)-arm galactosylated isomer were 2.4% and 0.9%, respectively, for HILIC(2-AB), and between 1.4% to 2.4% and 0.6% to 1.3%, respectively, for all other methods. The relative amount of doubly galactosylated, fucosylated species G2F was determined to be 9.6% for the HILIC(2-AB). This is nearly the same as found for CE-LIF(APTS-HR1) (**Table 2**). With HILIC(IAB), a slightly higher result was found (10.4%), and for the other methods slightly lower results (ranging from 8.2% to 9.5%) were observed. The sum of non-fucosylated species (G0+G1+G2), an important parameter for antibody effector function, averaged 8.4% for HILIC(2-AB), and nearly the same for HILIC(IAB) at 8.6%. All other methods featured lower amounts of non-fucosylated species, namely DSA-FACE(APTS) 7.1%, CCGE(ANTS) 6.5%, CE-LIF(APTS-HR2) 7.8% and HPAEC-PAD 7.1%. CE-LIF(APTS-HR1) could not detect G2, thus the lowest relative amount of non-fucosylated species (6.2%) was obtained with this method. However, since G2 is quantitated significantly below 1%, the influence of G2 on the afucosylation level is low.

The sum of monoantennary structures (structures lacking an N-acetylglucosamine G0F-N, G1F-N and G0-N) was found to average 0.9% for HILIC(2-AB). It must be noted, however, that G1F-N could not be fully separated. No monoantennary structures were resolved with CCGE(ANTS), and with CE-LIF(APTS-HR1) and DSA-FACE(APTS), only G0-N could be separated and quantified (relative amount 0.4% and 0.3%). The other methods showed similar relative amounts of monoantennary structures with 0.8% for CE-LIF(APTS-HR2), 0.9% for HILIC(IAB) and 1.3% for HPAEC-PAD.

For the M5 species, an average relative abundance of 1.5% was detected with HILIC(2-AB). Similar values were found for the other methods (ranging from 1.5% to 2.2%).

The sum of sialylated structures (G1FS, G2S1F and G2S2F) was found to be 1.0% with HILIC(2-AB). CE-LIF(APTS-HR1), CE-LIF(APTS-HR2), DSA-FACE(APTS) and HPAEC-PAD showed relative amounts ranging from 0.5% to 1.1%. HILIC(IAB) and CCGE(ANTS) exhibited the highest values, 1.7% to 1.9%, respectively.

### Analysis time and throughput

The information concerning analysis time and throughput is shown in **Table 3**. All methods employed were based on the analysis of glycans. The first step is always the release of the glycans, which is one of the most time-consuming steps. For the reference method, 4 h sample preparation time for 6 samples is needed, out of which about 1.5 h is hands-on time. In principle, there is not much difference between the methods. The fluorescence-based methods need labeling and clean-up steps. HPAEC-PAD was faster (1.5 h sample preparation) because no labeling step is required. The sample preparation for all methods can be automated; however, the DSA-FACE(APTS) method is the only method that is really a high-throughput method since 96 samples can be analyzed in parallel.

**Table 3.** Features of the methods

Method	Analysis time and throughput	Skills and investment needed	Required purity and sample amount
HILIC(2-AB) Reference Method	4 h sample preparation (1.5 h hands-on time); 10 h for 6 samples including separation; sample preparation can be automated	No special skills for the analyst needed; equipment: HPLC or UPLC with fluorescence detection; 2-AB labeling kits needed	No interference from contaminants observed; 200 μg mAb1
HILIC(IAB)	1.5 to 2 h sample preparation (1–1.5 h hands-on time) 8 h for 6 samples including separation; sample preparation expandable to 96 well plates; can be easily automated	No special skills for the analyst needed but must be properly trained in using the sample preparation system; equipment: HPLC or UPLC with fluorescence detection; 2-AB labeling kits needed	No interference from contaminants observed, 50 μg mAb1
CE-LIF(APTS-HR1)	24 h for 6 samples (5 h hands-on time); sample preparation and data evaluation can be automated but problems with robustness of the system	No special skills for the analyst needed but must be properly trained in using the sample preparation system; equipment: any CE-system with fluorescence detection; APTS labeling kits needed	No interference from contaminants observed; 300 μg mAb1
DSA-FACE(APTS)	For 96 samples 2–3 h hands-on time; optimally suited for high-throughput; 96 samples can be analyzed in parallel	No special skills for the analyst needed; DNA Analyzer with capillary technology needed, APTS labeling kits needed	No interference from contaminants observed; 5 μg mAb1
CE-LIF(APTS-HR2)	3 h for 6 samples (1–1.5 h hands-on time); electrophoretic analysis 35 min; total preparation time for 6 samples is 3.5 h; expandable to 96-well plates and easily automatable	No special skills for the analyst needed but must be properly trained in using the sample preparation system; equipment: any CE-system with fluorescence detection; APTS labeling kits needed	No interference from contaminants observed; 50 μg mAb1
CCGE(ANTS)	3 h for 6 samples (1.5–2 h hands-on time; total preparation and analysis time for 6 samples 3.5 h; expandable to 96 well plates and easily automatable	The analyst must be properly trained in using the sample preparation system; developmental CE is needed; special labware is needed; ANTS labeling kits needed	No interference from contaminants observed; 50 μg mAb1
HPAEC-PAD	Sample purification takes 30 min; separation time 1.5 h for one sample (hands-on time for 6 samples 1 h); no need for high-throughput for sample preparation	No special special skills for the analyst are needed; Investment: a HPLC system that is suited for the high-pH buffers and equipped with a pulsed amperometric detector	No interferences observed, but oligosaccharide and non-oligosaccharide contaminants might be of concern; 400 μg mAb1

### Skills and investments needed

As indicated in **Table 3**, no special skills were needed for any of the methods, but the analyst should be properly trained in the sample preparation and the instruments. For the fluorescence-based methods, a HPLC or CE system is required. Additionally, labeling kits must be purchased from time to time, which could contribute significantly to the cost.

### Required sample amount and purity

An overview is shown in **Table 3**. For the Reference Method and HILIC(IAB), interference from contaminants was not observed, but, since we used formulated bulk material as the sample, which is intrinsically very pure, the presence of contaminants from other samples that interfere with labeling could not be excluded. The same holds true for the CE-based methods. Contaminant interference for APTS and ANTS was not observed. For HPAEC-PAD, in principle, there could be problems with agents other than oligosaccharides that would also separate with a weak anion exchange column.

HILIC(2-AB)started with 200 μg of sample, and HILIC(IAB) with 50 μg. However, since the fluorescence detection for 2-AB is very sensitive, it should be possible to detect glycans in the femtomol quantities.^67^

The amount of sample was 300 μg for CE-LIF(APTS-HR1), 50 μg for CE-LIF(APTS-HR2) and 5 μg for DSA-FACE(APTS). These amounts were not optimized for the lowest amounts of mAb1. Adamczyk et al.^33^ estimated the detection limit of APTS-labeled glycans by CE-LIF to be 0.4 nM.

CCGE(ANTS) required an initial amount of 50 μg. However, as ANTS should have a comparable sensitivity to APTS, here also a detection limit of about 0.4 n*M* could be estimated. HPAEC-PAD started with 400 μg of sample; the detection limit for glycans, however, should be far below this amount.

## Discussion

Taken together the results obtained with all separation methods without mass spectrometric detection—with regard to the detection and quantitation of glycoforms—were very similar. With the exception of HPAEC-PAD, where the detection is based on amperometry, the other methods are based on fluorescence detection. The robust and comparable quantification of results is most likely due to the fact that only one fluorophore is added to the reducing end of the glycans. The detection with 2-AB-labeling is known to be very sensitive (femtomol quantities),^67^ but there may be a bias caused by partial glycan degradation during the labeling process, where the loss of the sialic acid could be of particular concern.^57^ We found no clear evidence for sialic acid degradation during labeling. A minor loss of sialic acid may occur with the standard 2-AB-labeling protocol (2 h at 65°C under acidic conditions) because only 1.0% sialic acid-containing glycans was found in comparison to 1.8% with InstantAB, where labeling takes place instantly at room temperature and neutral pH. The fluorophore used for 3 CE-based methods was APTS, where sialic-acid degradation may also occur during labeling. Additionally, electrokinetic injection was applied, and this might favor glycans with high mobility, *i.e*., sialylated species, since in CE separation the charged species migrate first. Such an effect was not seen in the study as the quantitative results of the main species for CE-based methods were similar to non-CE-based methods.

In general, it is a challenge to robustly quantitate sialylated species with CE-based methods, as they may co-migrate with other species. However, our study showed, in the case of the novel CCGE(ANTS) method, very similar results for the relative quantitation of sialylated species to those obtained with HILIC(IAB) method. This particularly good performance of CCGE(ANTS) for the analysis of sialylated species might be due to the label used (ANTS instead of APTS, which is used for the other CE methods) or to the chosen separation conditions. Notably, with regard to the main species, very similar results were obtained for all fluorescence detection-based methods, so there is no evidence for a labeling bias of different glycostructures.

HPAEC-PAD showed slight differences in galactosylation levels compared to the other methods. Existing scientific literature suggests that the response for different glycostructures varies in HPAEC-PAD, but that the effect is low. Increasing glycan size and sialylation is believed to cause reduction in PAD response.^68,69^ This might account for the small differences for G0F and the sialylated species.

As with many methods, there are drawbacks with those discussed here. With HILIC(2-AB), G1F-N could not be separated and in general the method is relatively time-consuming. As stated before, there could be an underestimation in sialic acid quantitation due to a bias in the labeling process. Concerning the CE-based methods, as mentioned before, the loss of sialic acid may be of concern and some peaks co-migrate. Regarding the HPAEC method, there is the concern that one has to apply response factors for the different glycostructures, as discussed before. It is always a good practice to use 2 different methods to circumvent these drawbacks.

In summary, all 7 methods showed excellent performance for accuracy, precision and separation, and are well suited for the purpose of analyzing Fc-glycosylation of IgG1. The relative quantitation for the individual glycan species were comparable. In principle, all methods could be used as release methods, and validating them should be no problem. In our hands, the Reference Method, HILIC(2-AB), is optimally suited for release. The method found to be best suited for high throughput was DSA-FACE(APTS), where 96 samples can be analyzed in parallel. If all glycan structures of a mAb must be quantified, the use of 2 methods in parallel is advised. All methods in the study exhibited excellent standard deviations and low day-to-day variability. The situation is more diverse with sialylated species, as methods with rapid-reductive-amination labeling detected higher amounts. All methods described and tested here could in principle also be applied for Fc-fusion proteins, bispecific antibodies and glycoengineered antibodies as well as other glycoproteins. However, when site-specific information is essential, mass spectrometry-based methods might be more useful.^1,3,55,60,70^ In the second part of our report, the mass spectrometry-based methods will be presented and an overall comparison between all methods will be given.

## Materials and Methods

MAb1 was produced in a CHO cell line, and purified by the Downstream Processing Group at Roche Diagnostics GmbH.

### HILIC(2-AB) (The Reference Method)

MAb1 (200 μg, 350 μl) was buffer exchanged with the aid of Nanosep® centrifugal devices (Pall, USA) to ammonium formate buffer (10 mM, pH 8.6). N-glycosidic-bound oligosaccharides were released by incubating 48-μl samples with 2 μl PNGase F (500,000 U/ml, New England Biolabs) at 45°C for 1 h. Released glycans were labeled with 2-AB at 65°C for 2 h (Glyko® Signal 2-AB Labeling Kit, ProZyme). Excess 2-AB was removed using HyperSep-96 Diol cartridges (Thermo) with a vacuum station. Labeled glycans were washed with 96% acetonitrile, eluted from the cartridges and analyzed by HILIC-UHPLC using a Waters BEH Glycan Separation Technology column (2.1 × 150 mm, 1.7 μm) on a Dionex RSLC Ultimate 3000RS or a Waters ACQUITY UPLC® system. A 45-min acetonitrile gradient was applied and fluorescence signals were detected at 420 nm (excitation at 330 nm). Peaks were integrated automatically according to pre-defined parameters with the software Chromeleon© and relative glycan compositions were calculated.

### HILIC(IAB)

MAb1 (50 μg) was prepared using GlykoPrep® Rapid N-Glycan Sample Preparation (GS96-RX–GlykoPrep Digestion Module); the sample was mixed with Denaturation Reagent and incubated at room temperature for 5 min. The denatured protein mixture was applied to the RX Cartridges, where the protein is immobilized and deglycosylated with PNGase F for 30 min on a heatblock set to 50°C. The released N-glycans were eluted and immediately labeled at room temperature (without incubation) with GlykoPrep InstantAB™ (GS96-LB–GlykoPrep InstantAB Labeling Module). Labeled N-glycans were then cleaned up using CU Cartridges to remove excess labeling reagents. InstantAB-labeled glycans were then analyzed by UPLC-FLR using a Waters BEH-Glycan Separations Technology column (1.7 μm, 150 × 2.1 mm, part number 180064742) with an increasing ammonium formate gradient (mobile phase A: Acetonitrile; mobile phase B: 100 mM Ammonium formate at pH 4.4) over 60 min. An injection volume of 1 μl aqueous and a column temperature of 35°C were used; glycans were detected at a wavelength of 330 nm with an excitation wavelength of 278 nm. Peaks were integrated using Empower® software (Waters Corp.), and relative glycan compositions were calculated.

### CE-LIF(APTS-HR1)

MAb1was diluted to approximately 10 mg/ml (30 μl) with product formulation buffer using a Microcon-30 concentrator (Amicon). PNGase F diluted in reaction buffer (50 mM sodium succinate pH 5.5) was added, and the sample was incubated for approximately 15 h at 37°C. The deglycosylated protein was heated and precipitated by centrifugation. The supernatant was dried and reconstituted in an excess solution of 15 μl of acidic APTS (ProZyme) (5 mg in 0.5 ml of 15% v/v glacial acetic acid) and 5 μl of 1 *M* sodium cyanoborohydride in tetrahydrofurane (Aldrich). This solution was heated at 55°C for 2 h. The solution was diluted with water to a final volume of 250 μl.

CE-LIF experiments were performed using a Beckman Coulter PA800 *plus* Pharmaceutical Analysis System with LIF detection (ex: 488 nm; and em: 520 nm). Separation was performed with Beckman eCAP neutral capillaries (60 cm total length; 50 cm effective length; 50 μm ID; 360 μm OD; Beckman Coulter); running buffer was a 50/50 mixture of carbohydrate separation buffer and DNA gel buffer (Beckman Coulter); an applied voltage of -30 kV. Capillaries were kept at 20°C and flushed with running buffer prior to each analysis. No additional conditioning was used. Injection was performed hydrodynamically at 0.5 psi for 10 s.

Peaks were integrated automatically according to pre-defined parameters with the software 32-Karat© (% corrected peak area) and relative glycan compositions were calculated.

### DSA-FACE(APTS)

MAb1 (5 μg) was transferred to AcroPrep^TM^ Advance 96-Well Filter Plates 30 K Omega from Pall and water was added to give a final volume of 300 μl. The plates were centrifuged 3 times after addition of 300 μl of water for 5 times with 1500 × g.

Samples were reconstituted in 50 μl of water containing 1 μl of PNGase F (250 U of enzyme were dissolved in 250 μl water). Filter plates were sealed and the samples were directly incubated on the filter at 37°C overnight. The released glycans were separated from IgG via the filter plates by centrifugation for 5 min at 1500 × g into 96-well receiver plates (ProZyme). Samples were dried by vacuum centrifugation.

Labeling was performed with the GlykoPrep® Rapid-Reductive-Amination APTS Labeling Module for 96-well plates (ProZyme, GS96-APTS), consisting of reductant solution, APTS solution and APTS catalyst solution. For 96 samples, typically 104 μl of reductant, 260 μl of APTS catalyst and 104 μl of APTS solution were mixed. Dried glycans where reconstituted in 4.5 μl of the prepared APTS-labeling master mix, the plates were sealed and labeling performed with light excluded for 4 h at 50°C. Clean up after labeling was performed with GlykoPrep Clean Up (CU) Cartridges (GS96-C2, ProZyme). Then 20 ml of 5× APTS sample loading buffer (ProZyme) was filled up to 100 ml with acetonitrile. Samples were then diluted in 200 μl APTS sample loading buffer with thorough mixing and subsequently loaded onto the CU Cartridges using 3 min centrifugal force at 300 × g followed for 1 min at 1000 × g. Then CU Cartridges were washed with 2 times 200 μl of APTS sample loading buffer by centrifugation for 3 min at 300 × g to remove excess dye and labeling side products. Finally samples were eluted with 2 times 50 μl of water by centrifugation for 3 min at 1000 × g.

For 96 samples 1250 μl Hi-Di Formamide (Applied Biosystems product code 4311320) was mixed vigorously with 3.5 μl of Basepair Size Standard (Applied Biosystems 500 Rox Size Standard product code 401734). Cleaned up samples were diluted 1:20 with water. Prior to analysis 2 μl of diluted samples were then mixed with 10 μl HI-DI Formamide Basepair Size Standard mixture.

Analyses were performed with a 48-capillary array (50-cm length; filled with Pop-7™ Polymer (Applied Biosystems). Injection was performed with an injection voltage of 3 kV for 15 sec; separations were performed at 15 kV over a run time of 1800 s. Data analysis was performed by an in-house-developed Matlab application. The software normalized the migration time on the internal base pair standard by a regression function of 2nd polynomial order. The area of the assigned peaks was determined and the relative area of the glycans were calculated.

### CE-LIF(APTS-HR2)

MAb1 (50 μg) was prepared using GlykoPrep® Rapid N-Glycan Sample Preparation (GS96-RX–GlykoPrep Digestion Module); the sample was mixed with Denaturation Reagent (ProZyme) and then incubated at room temperature for 5 min. The denatured protein mixture is applied to the RX Cartridges, where the protein is immobilized and deglycosylated with PNGase F for 30 min on a heat block set to 50°C. The released N-glycans are eluted and immediately treated with Finishing Reagent and incubated for 10 min on a 50°C heat block to convert the released glycans to the reduced, aldehyde form; N-glycans are then dried in a centrifugal evaporator, followed by labeling with GlykoPrep Rapid-Reductive-Amination™ APTS (GS96-APTS–Reductive-Amination APTS Labeling Module). Labeled N-glycans were purified using CU Cartridges to remove excess labeling reagents. APTS-labeled glycans were analyzed by CE-LIF with a Beckman Coulter PA800 *plus* Pharmaceutical Analysis System using a Beckman-Coulter N-CHO capillary (50 μm inner diameter, 60 cm total length—50 cm effective, part number 477601) with a separation voltage of 20 kV (reversed polarity) over 35 min. The buffer used was a 1:1 mixture of N-Linked Carbohydrate Separation Gel Buffer and eCAP™ ds DNA 1000 Gel. An injection protocol of 2 psi for 10 seconds and a capillary temperature of 20°C were used. Glycans were detected at a wavelength of 520 nm with an excitation wavelength of 488 nm. Peaks were integrated using Empower*®* software (Waters Corp) and relative glycan compositions were calculated.

### CCGE(ANTS)

MAb1 (50 μg) was prepared using GlykoPrep® Rapid N-Glycan Preparation (GS96-RX–GlykoPrep Digestion Module); the antibody is mixed with Denaturation Reagent and then incubated at room temperature for 5 min. The denatured protein mixture is applied to the RX Cartridges, where the protein is immobilized and deglycosylated with PNGase F for 30 min on a heatblock set to 50°C. The released N-glycans are eluted, immediately treated with Finishing Reagent and incubated for 10 min on a 50°C heatblock to convert the released glycans to the reduced, aldehyde form. Reduced glycans are then dried in a centrifugal evaporator. Dried glycans are labeled with a developmental labeling kit utilizing (ANTS) as the fluorescent tag. Labeled N-glycans are cleaned up using CU Cartridges to remove excess labeling reagents. ANTS-labeled glycans are analyzed by CE-FLR using a developmental cartridge-based capillary gel electrophoresis system (capillary: 75 μm inner diameter, 15.5 cm total length—11.5 cm effective) with a separation voltage of 6 kV (reversed polarity) over 4 min. The buffer used contained a gel matrix. An injection protocol of 3 kV for 10 s was used and the capillary was operated at ambient temperatures; glycans were detected at a wavelength of 530 nm with an excitation wavelength of 420 nm. Peaks were integrated using Empower® software (Waters Corp) and relative glycan compositions were calculated.

### HPAEC-PAD

MAb1 (400 μg) was transferred to NAP5^©^-columns (GE-Healthcare 17–0853–02) and buffered with 10 m*M* sodium phosphate pH 7.2 and concentrated to a final volume of 50 μL. Subsequently the sample was incubated with 2 Units of N-Glycosidase F (Roche 1 365 193) for 16 h at 37°C. Vivaspin concentrators (0.5 ml; 10 kD; Sartorius, (Göttingen, Germany) were used to separate the glycans from residual protein. The remaining solution was washed 2 times with 30 μl of water and diluted with water to give a final volume of 140 μl. HPAEC-PAD experiments were performed using a BioLC equipped with a CarboPAc PA200 column (ThermoFisher Scientific, Bremen, Germany). Mobile phase A was 50 m*M* NaOH and mobile phase B was 50 m*M* NaOH, 200 m*M* sodium acetate. The glycans were eluted using a gradient to give 3% Eluent B after 25 min, 20% Eluent B after 55 min and 70% Eluent B after 90 min. A flow rate of 0.5 mL/min was used; the injection volume was 10 μl. Detection was by means of pulsed amperometric detection.

Peaks were integrated automatically according to pre-defined parameters with the software Chromeleon©, and relative glycan compositions were calculated.
